# Analysis of Risk Factors and Development of a Predictive Nomogram for Bronchiolitis Obliterans in Children With Severe Adenovirus Pneumonia: A Retrospective Study

**DOI:** 10.1155/carj/2606203

**Published:** 2026-07-20

**Authors:** Gang Chen, Xing Liu, Xiuchang Ma, Jinhuan Wu, Yaowen Liang, Yuchen Du, Xiaoyu Song, Wenxian Qian, Apeng Chen, Changhua Yi, Man Tian

**Affiliations:** ^1^ Department of Respiratory, Children’s Hospital of Nanjing Medical University, Nanjing, Jiangsu, China, njmu.edu.cn; ^2^ Department of Pediatrics, The First People’s Hospital of Lianyungang, Xuzhou Medical University Affiliated Hospital of Lianyungang (Lianyungang Clinical College of Nanjing Medical University), Lianyungang, Jiangsu, China, lygyy.com.cn; ^3^ Clinical Research Centre, The Second Hospital of Nanjing, Affiliated Hospital to Nanjing University of Chinese Medicine, Nanjing, Jiangsu, China, njmu.edu.cn; ^4^ School of Clinical and Basic Medical Sciences, Shandong First Medical University & Shandong Academy of Medical Sciences, Jinan, Shandong, China, sdfmu.edu.cn; ^5^ Nanjing Key Laboratory of Pediatrics, Children’s Hospital of Nanjing Medical University, Nanjing, Jiangsu, China, njmu.edu.cn

**Keywords:** bronchiolitis obliterans, children, logistic regression analysis, risk factors, severe adenovirus pneumonia

## Abstract

**Background:**

Bronchiolitis obliterans (BO) is a rare chronic pulmonary condition in pediatric patients, characterized by irreversible fibrotic constriction of the small airways. This study aims to analyze the risk factors for BO caused by severe adenovirus pneumonia (SAP) and to develop a nomogram model for personalized prediction.

**Methods:**

We retrospectively analyzed 251 children with SAP admitted to the Children’s Hospital of Nanjing Medical University between January 2017 and March 2024. Among them, 77 were classified into the BO group and 174 into the non‐BO group. A predictive nomogram was developed based on independent risk factors identified through univariate analysis, least absolute shrinkage and selection operator (LASSO) regression, and multivariate logistic regression.

**Results:**

Multivariable analysis identified the following independent risk factors for BO: wheezing (odds ratio [OR] = 4.78; 95% confidence interval [CI], (2.00∼11.40); *p* < 0.001), large lobar consolidation (OR = 8.53; 95% CI, 3.63∼20.02; *p* < 0.001), length of hospital stay (OR = 1.20; 95% CI, 1.08–1.33; *p* < 0.001), and duration of fever (OR = 1.20; 95% CI, 1.09–1.32; *p* < 0.001). Mechanical ventilation showed a trend toward increased risk but did not reach statistical significance (OR = 5.35; 95% CI, 0.74–38.56; *p* = 0.096). The nomogram demonstrated excellent discriminative ability, with an area under the curve of 0.90 (95% CI: 0.85–0.94). Calibration curves indicated good agreement between predicted and observed outcomes. Decision curve analysis showed a net clinical benefit within a clinically relevant threshold probability range of 0.10–0.70.

**Conclusions:**

A nomogram incorporating five independent risk factors—wheezing, duration of fever, length of hospital stay, mechanical ventilation, and large lobar consolidation—was developed to predict BO risk in children with SAP.

## 1. Introduction

Bronchiolitis obliterans (BO) is a chronic and irreversible obstructive pulmonary syndrome characterized by inflammatory injury to the small airways [1, 2]. Histopathologically, the condition presents with subepithelial inflammation and fibrotic narrowing of the bronchioles [3]. The primary clinical manifestations of BO include recurrent or persistent cough, wheezing, shortness of breath, and exercise intolerance. These symptoms reflect a disease that often carries a poor prognosis in children, frequently leading to significant deterioration in pulmonary function [4]. Regarding etiology, BO is associated with severe lower respiratory tract infections (LRTIs), hematopoietic stem cell transplantation, autoimmune disorders, and exposure to toxic substances [2, 5]. In pediatric patients, the predominant cause of BO is severe LRTIs, also referred to as post‐infectious bronchiolitis obliterans (PIBO) [5].

Adenovirus (ADV) is a common pathogen of community‐acquired pneumonia in children, with 20.6%–37.9% of cases potentially progressing to severe adenovirus pneumonia (SAP) [6, 7]. SAP exhibits a rapid clinical progression and is associated with a poor prognosis, frequently accompanied by multiorgan dysfunction [8], with a mortality rate of approximately 6.88% [9]. Approximately 14%–60% of survivors experience long‐term sequelae, among which BO is a common complication [10]. Even years post‐discharge after treatment for SAP, many children continue to suffer from persistent cough, increased susceptibility to pulmonary infections requiring hospitalization, and reduced exercise tolerance [10]. Commonly employed therapies—systemic corticosteroids, macrolide antibiotics, leukotriene‐receptor antagonists, and intravenous immunoglobulin (IVIG)—have proven largely ineffective at substantially improving pulmonary function [5]. This limited efficacy may be attributable to late diagnosis of BO, by which time irreversible pulmonary fibrosis has often already developed. Therefore, identifying risk factors for the development of BO following SAP is critically important. Early recognition, diagnosis, and intervention in high‐risk children may improve prognosis and quality of life in those affected by BO. Previous studies have identified several independent risk factors for post‐ADV BO in children, including extended length of duration of fever, higher lactic dehydrogenase (LDH) levels, peripheral blood neutrophil percentage, acute respiratory failure, multifocal pneumonia, hypercapnia, invasive mechanical ventilation, and ICU admission [11–13]. However, studies on risk factors for BO—particularly following SAP—remain scarce, underscoring the need for further investigation. This study retrospectively analyzed the clinical, laboratory, and radiological variables of pediatric patients with SAP, comparing those who developed BO with those who did not. A predictive model for BO was developed to facilitate early identification of high‐risk cases, thereby enabling timely interventions and potentially improving clinical outcomes.

## 2. Materials and Methods

### 2.1. Patients and Case Definition

**FIGURE 1:**
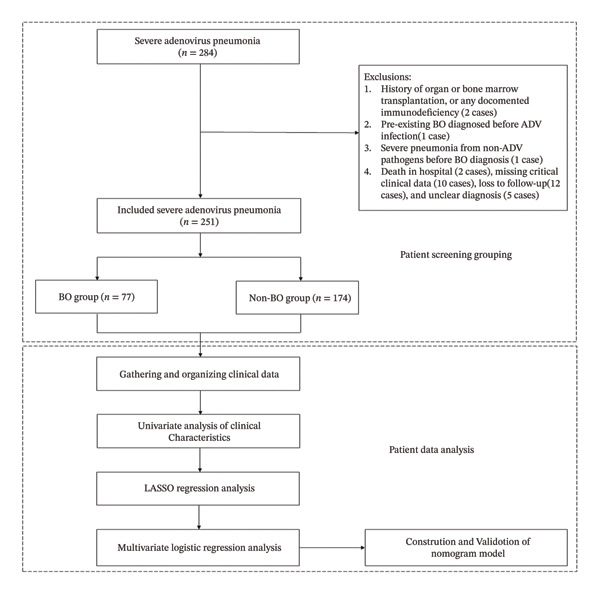
Flowchart of the study process.

We performed a retrospective analysis of children diagnosed with SAP and hospitalized at the Children’s Hospital of Nanjing Medical University between January 2017 and March 2024. The diagnosis and exclusion of all patients were determined by two experienced pediatric pulmonologists. The detailed patient enrollment and grouping process is summarized in Figure 1.

Inclusion criteria include the following: (1) age 28 days to 14 years; (2) evidence of pneumonia on clinical and radiological evidence, supported by at least one positive laboratory finding—ADV nucleic acid detected in a nasopharyngeal swab or ADV sequences identified in bronchoalveolar‐lavage fluid via next‐generation sequencing; (3) fulfillment of the diagnostic criteria for severe pneumonia.

Exclusion criteria include the following: (1) history of organ or bone marrow transplantation, or any documented immunodeficiency; (2) pre‐existing BO diagnosed before ADV infection; (3) previous severe pneumonia caused by pathogens other than ADV before the diagnosis of BO; (4) incomplete or missing key clinical data precluding reliable statistical analysis (as defined in the Data Collection section).

Severe pneumonia was defined in accordance with the management guidelines for community‐acquired pneumonia established by the American Thoracic Society [14]. The major criteria comprise: the necessity for invasive mechanical ventilation; fluid‐refractory shock; an acute requirement for noninvasive positive pressure ventilation; and hypoxemia necessitating a fraction of inspired oxygen (FiO_2_) that exceeds the concentration or flow rate feasible in a general care setting. The minor criteria include: a respiratory rate surpassing the age‐specific thresholds recommended by the World Health Organization; apnea; increased labored breathing, as manifested by retractions, dyspnea, nasal flaring, or grunting; a PaO_2_/FiO_2_ ratio of less than 250; multilobar infiltrates; altered mental status; hypotension; the presence of pleural effusion; comorbid conditions; and unexplained metabolic acidosis. The diagnosis of PIBO in the pediatric patients was established based on the following criteria [15]: (1) a history of relatively severe prior LRTIs, most notably those attributed to ADV, *Mycoplasma pneumoniae* (MP), and measles virus; (2) persistent or recurrent clinical manifestations of airway obstruction following pulmonary infection, including wheezing, coughing, dyspnea, and exercise intolerance, accompanied by persistent auscultatory findings (e.g., wheezing and moist rales) in the affected areas for > 6 weeks, and a poor or absent bronchodilator response; (3) high‐resolution computed tomography (HRCT) mainly shows a mosaic attenuation pattern and/or expiratory air trapping (often accompanied by vascular pruning/hypoperfusion), and may be accompanied by bronchiectasis and bronchial wall thickening; (4) pulmonary function tests revealed obstructive or mixed ventilatory impairment, characterized by either an FEV_1_/FVC ratio of less than 80% or a predicted FEV_1_ below 80%, with bronchodilator responses predominantly negative; (5) the exclusion of other chronic pulmonary conditions that could cause wheezing and coughing, such as bronchial asthma, congenital bronchopulmonary dysplasia, tuberculosis, and cystic fibrosis.

Large lobar consolidation was characterized as consolidation affecting more than 2/3 of a single lobe or involving multiple lobes, potentially accompanied by pleural effusion and/or focal bronchiolitis [16]. Diffuse bronchiolitis was characterized by bronchiolar abnormalities, including centrilobular nodules, bronchiolar wall thickening, reticulonodular opacities, or branching linear opacities, distributed diffusely within a single lobe or bilaterally, and occupying at least four‐fifths of a lobe, with or without atelectasis resulting from mucous plugging. The above two radiological findings were independently reviewed and analyzed by two experienced pediatric radiologists, with any disagreements resolved by consensus. Respiratory failure was defined as the inability of the respiratory system to maintain adequate gas exchange, resulting in hypoxia (PaO_2_ < 60 mmHg) with or without hypercapnia (PaCO_2_ > 50 mmHg), as confirmed by arterial blood gas analysis.

### 2.2. Data Collection

A total of 251 pediatric patients with SAP were enrolled. Their complete inpatient electronic medical records were systematically reviewed. The following key clinical data were extracted and analyzed: (1) baseline demographic characteristics, including age, sex, and the presence of atopic constitution (a history of atopic dermatitis, allergic rhinitis, asthma, elevated total IgE (> 200 IU/mL), or positive specific IgE to common allergens); (2) clinical features, including the duration of fever, length of hospital stay, occurrence of wheezing during illness, underlying medical, occurrence of wheezing during illness, underlying medical, occurrence of wheezing during illness, underlying medical conditions, and systemic complications; (3) laboratory results, comprising complete blood count, C‐reactive protein levels, coagulation profile, liver enzyme levels, cardiac biomarkers, and serum immunoglobulin levels; (4) imaging findings, such as large lobar consolidation, diffuse bronchiolitis, and pleural effusion; (5) in‐hospital treatments, including administration of IVIG or systemic corticosteroids, and use of bronchoscopy. All patients were followed for at least 18 months after discharge through telephone and WeChat communication to assess the persistence or recurrence of respiratory symptoms and signs, their duration, and to review postdischarge chest CT and pulmonary function test results. The median follow‐up time was 42 months (range, 18–69 months). Based on the definitive diagnosis of BO during the follow‐up period, patients were categorized into either the BO group or the non‐BO group.

### 2.3. Ethics Approval and Consent to Participate

Conducted in accordance with the Declaration of Helsinki, this study was approved by the Medical Ethics Committee of the Children’s Hospital of Nanjing Medical University (Approval No. 20250919‐1) with a waiver of informed consent. This waiver was granted based on the retrospective nature of the research and the minimal risk to the participants. All patient data were anonymized before analysis to protect confidentiality.

### 2.4. Statistical Analysis

All statistical analyses were performed using R software, version 4.3.0 (R Foundation for Statistical Computing). Continuous variables with normal distribution are expressed as mean ± standard error (SE) and were compared between groups using the independent samples *t*‐test. Non‐normally distributed data are expressed as median [P25, P75] and were compared with the Mann–Whitney *U* test. Categorical variables are reported as frequencies and percentages, and between‐group differences were assessed with the chi‐square (*χ* ^2^) test. Given the very low proportion of missing data (< 1%), complete‐case analysis was utilized for the multivariate modeling. To identify risk factors for BO development following SAP in children, least absolute shrinkage and selection operator (LASSO) regression was applied with the “glmnet” package. Variables selected by LASSO were then included in a multivariate logistic regression model to determine independent risk factors. A nomogram was constructed and internally validated with the “rms” package; its discriminative performance was evaluated via receiver operating characteristic (ROC) curves generated with the “pROC” package. Clinical utility was assessed with decision curve analysis (DCA) using the “dcurves” package. In univariate analyses, a two‐sided *p* < 0.05 was considered statistically significant; in the multivariate logistic regression model, significance was set at *p* < 0.10.

## 3. Results

### 3.1. Comparison of Clinical Characteristics Between the BO and Non‐BO Groups

A total of 251 children with SAP were included in the study. Among them, 77 cases (30.68%) were classified into the BO group and 174 (69.32%) into the non‐BO group. Age distribution differed significantly between the two groups (*χ* ^2^ = 12.82; *p* = 0.002). The BO group had a significantly higher proportion of children under 12 months of age (24.68% vs 12.07%) and under 24 months of age (27.27% vs 16.67%) compared to the non‐BO group. Conversely, the non‐BO group included a higher proportion of children over 24 months of age (71.26% vs. 48.05%). Of the 251 SAP patients, 158 (62.95%) were male; however, no significant difference in sex distribution was observed between the BO and non‐BO groups (Table 1).

**TABLE 1:** Univariate comparison of clinical features and laboratory markers between two groups.

Variables	Total (*n* = 251)	Non‐BO group (*n* = 174)	BO group (*n* = 77)	Statistic	*p*
Age (months), *n* (%)				*χ* ^2^ = 12.82	**0.002**
≤ 12 month	40 (15.94)	21 (12.07)	19 (24.68)		
≤ 24 month	50 (19.92)	29 (16.67)	21 (27.27)		
> 24 month	161 (64.14)	124 (71.26)	37 (48.05)		
Sex, male, *n* (%)	158 (62.95)	108 (62.07)	50 (64.94)	*χ* ^2^ = 0.19	0.665
Length of hospital stay, M (*Q*_1_, *Q*_3_)	9.00 (7.00, 13.00)	8.00 (7.00, 10.00)	13.00 (10.00, 17.00)	*Z* = −7.22	**< 0.001**
Peak temperature, M (*Q*_1_, *Q*_3_)	39.60 (39.00, 40.00)	39.50 (39.00, 40.00)	39.70 (39.30, 40.10)	*Z* = −2.58	**0.010**
Duration of fever, M (*Q*_1_, *Q*_3_)	10.00 (7.00, 13.00)	9.00 (6.00, 11.00)	13.00 (9.00, 16.00)	*Z* = −6.08	**< 0.001**
Wheezing, *n* (%)	76 (30.28)	38 (21.84)	38 (49.35)	*χ* ^2^ = 19.14	**< 0.001**
PICU admission, *n* (%)	47 (18.73)	15 (8.62)	32 (41.56)	*χ* ^2^ = 38.05	**< 0.001**
Underlying disease, *n* (%)	103 (41.04)	67 (38.51)	36 (46.75)	*χ* ^2^ = 1.50	0.221
Atopic, *n* (%)	55 (21.91)	26 (14.94)	29 (37.66)	*χ* ^2^ = 16.10	**< 0.001**
Imaging findings, *n* (%)					
Large lobar consolidation	114 (45.42)	53 (30.46)	61 (79.22)	*χ* ^2^ = 51.20	**< 0.001**
Diffuse bronchiolitis	12 (4.78)	4 (2.30)	8 (10.39)	*χ* ^2^ = 6.00	**0.014**
Pleural effusion	28 (11.16)	19 (10.92)	9 (11.69)	*χ* ^2^ = 0.03	0.858
Co‐infection *n* (%)					
Bacteria,	77 (30.68)	53 (30.46)	24 (31.17)	*χ* ^2^ = 0.01	0.911
Virus	70 (27.89)	49 (28.16)	21 (27.27)	*χ* ^2^ = 0.02	0.885
Mycoplasma pneumoniae	102 (40.64)	78 (44.83)	24 (31.17)	*χ* ^2^ = 4.13	**0.042**
Complication *n* (%)					
Respiratory failure	33 (13.15)	11 (6.32)	22 (28.57)	*χ* ^2^ = 23.14	**< 0.001**
Heart failure	29 (11.55)	9 (5.17)	20 (25.97)	*χ* ^2^ = 22.60	**< 0.001**
Neurological impairment	8 (3.19)	3 (1.72)	5 (6.49)	*χ* ^2^ = 2.54	0.111
Sepsis	15 (5.98)	6 (3.45)	9 (11.69)	*χ* ^2^ = 5.07	**0.024**
Treatment, *n* (%)					
Glucocorticoid	152 (60.56)	94 (54.02)	58 (75.32)	*χ* ^2^ = 10.14	**0.001**
IVIG	93 (37.05)	47 (27.01)	46 (59.74)	*χ* ^2^ = 24.52	**< 0.001**
Mechanical ventilation	17 (6.77)	2 (1.15)	15 (19.48)	*χ* ^2^ = 28.41	**< 0.001**
Bronchoalveolar lavage	102 (40.64)	77 (44.25)	25 (32.47)	*χ* ^2^ = 3.07	0.080
HGB, mean ± SD	119.86 ± 12.42	121.67 ± 12.45	115.77 ± 11.43	*t* = 3.55	**< 0.001**
Tcell, mean ± SD	55.34 ± 13.96	57.05 ± 13.64	51.49 ± 13.99	*t* = 2.95	**0.003**
CRP, M (*Q*_1_, *Q*_3_)	12.00 (8.00, 30.66)	14.50 (8.00, 31.92)	10.40 (8.00, 21.07)	*Z* = −0.95	0.342
WBC, M (*Q*_1_, *Q*_3_)	13.07 (9.82, 17.66)	13.18 (9.71, 17.55)	12.75 (10.25, 18.89)	*Z* = −0.19	0.850
NEUT, M (*Q*_1_, *Q*_3_)	7.15 (4.46, 11.42)	7.17 (4.54, 11.31)	6.90 (4.21, 12.29)	*Z* = −0.09	0.931
RBC, M (*Q*_1_, *Q*_3_)	4.44 (4.14, 4.67)	4.46 (4.18, 4.67)	4.37 (4.02, 4.66)	*Z* = −1.09	0.277
PT, M (*Q*_1_, *Q*_3_)	11.70 (10.90, 12.70)	11.70 (11.00, 12.70)	11.70 (10.80, 12.60)	*Z* = −0.25	0.801
INR, M (*Q*_1_, *Q*_3_)	1.03 (0.96, 1.12)	1.03 (0.97, 1.11)	1.03 (0.95, 1.13)	*Z* = −0.22	0.828
APTT, M (*Q*_1_, *Q*_3_)	31.60 (28.40, 36.00)	31.15 (28.13, 35.38)	32.70 (29.50, 37.80)	*Z* = −2.22	**0.026**
FIB, M (*Q*_1_, *Q*_3_)	3.22 (2.68, 3.79)	3.25 (2.71, 3.86)	3.03 (2.63, 3.57)	*Z* = −1.84	0.066
TT, M (*Q*_1_, *Q*_3_)	14.90 (13.70, 16.40)	14.70 (13.40, 16.28)	15.50 (14.00, 16.90)	*Z* = −1.98	**0.048**
D dimer, M (*Q*_1_, *Q*_3_)	784.00 (457.00, 1404.00)	805.00 (462.00, 1300.75)	675.00 (433.00, 1797.00)	*Z* = −0.09	0.927
FDP, M (*Q*_1_, *Q*_3_)	5.33 (2.96, 9.34)	5.35 (3.20, 8.17)	5.11 (2.87, 11.13)	*Z* = −0.00	0.996
ALT, M (*Q*_1_, *Q*_3_)	20.00 (13.00, 33.50)	20.00 (13.00, 31.00)	22.00 (16.00, 40.00)	*Z* = −1.50	0.133
AST, M (*Q*_1_, *Q*_3_)	40.00 (28.50, 62.00)	38.00 (25.00, 49.00)	55.00 (35.00, 76.00)	*Z* = −4.58	**< 0.001**
LDH, M (*Q*_1_, *Q*_3_)	543.00 (406.00, 732.50)	513.50 (381.50, 668.75)	625.00 (460.00, 972.00)	*Z* = −3.77	**< 0.001**
CK, M (*Q*_1_, *Q*_3_)	62.00 (42.00, 121.50)	55.00 (40.00, 96.00)	102.00 (58.00, 179.00)	*Z* = −4.04	**< 0.001**
CKMB, M (*Q*_1_, *Q*_3_)	31.00 (26.00, 40.50)	30.50 (25.00, 40.00)	33.00 (27.00, 42.00)	*Z* = −1.30	0.193
IGG, M (*Q*_1_, *Q*_3_)	9.53 (6.91, 12.55)	9.59 (7.18, 12.57)	9.00 (6.43, 12.21)	*Z* = −1.22	0.221
IGM, M (*Q*_1_, *Q*_3_)	1.57 (1.06, 2.46)	1.66 (1.10, 2.55)	1.39 (1.02, 2.09)	*Z* = −1.59	0.113
CD4, M (*Q*_1_, *Q*_3_)	29.13 (22.31, 37.08)	29.97 (23.41, 37.36)	26.76 (18.35, 35.82)	*Z* = −2.40	**0.016**
CD8, M (*Q*_1_, *Q*_3_)	22.09 (18.49, 28.07)	23.01 (19.27, 28.76)	21.04 (17.35, 23.92)	*Z* = −2.03	**0.042**
CD4CD8, M (*Q*_1_, *Q*_3_)	0.60 (0.29, 1.13)	0.62 (0.28, 1.21)	0.57 (0.32, 1.08)	*Z* = −0.72	0.470
NK, M (*Q*_1_, *Q*_3_)	7.80 (4.92, 12.31)	7.82 (5.20, 12.33)	7.78 (4.11, 11.72)	*Z* = −0.99	0.321
B cell, M (*Q*_1_, *Q*_3_)	28.56 (20.76, 40.28)	27.02 (19.28, 38.34)	33.96 (25.06, 43.10)	*Z* = −2.99	**0.003**

*Note: t*, *t*‐test; *Z*, Mann–Whitney test; *χ* ^2^, chi‐square test; M, median; *Q*_1_, 1st quartile; *Q*_3_, 3st quartile; HGB. hemoglobin; NEUT, neutrophil count; FIB, fibrinogen; ALT, alanine aminotransferase; AST, aspartate aminotransferase; LDH, lactate dehydrogenase; CKMB, creatine kinase‐MB; NK, natural killer cell. Values in bold indicate *p* < 0.05 versus baseline.

Abbreviations: APTT, activated partial thromboplastin time; CK, creatine kinase; CRP, C‐reactive protein; FDP, fibrinogen degradation products; INR, international normalized ratio; PT, prothrombin time; RBC, red blood cell; SD, standard deviation; TT, thrombin time; WBC, white blood cell.

Additionally, the BO group was characterized by significantly longer hospital stays, higher peak temperatures, prolonged duration of fever, and higher rates of PICU admission, wheezing, atopic history, MP co‐infection, respiratory failure, heart failure, and sepsis (all *p* < 0.05). In contrast, the presence of underlying diseases, co‐infection with other bacteria or viruses, and neurological impairment did not differ significantly between the groups (all *p* > 0.05) (Table 1).

Upon admission, laboratory indices and imaging findings for patients with SAP were documented, as presented in Table 1. The BO group demonstrated significantly lower hemoglobin levels compared to the non‐BO group (*p* = 0.001). Furthermore, the BO group showed elevated median values for activated partial thromboplastin time and thrombin time (*p* < 0.05). Tissue injury markers, including aspartate aminotransferase, LDH, and creatine kinase (CK), were significantly higher in the BO group (*p* < 0.001). Immunological markers revealed a decrease in CD4^+^ T‐cell counts, along with increased total T‐cell and B‐cell counts in the BO group (*p* < 0.05). No significant differences were observed between groups in C‐reactive protein, white blood cell count, neutrophil Percentage, prothrombin time, international normalized ratio, fibrinogen, D‐dimer, fibrin degradation products, alanine aminotransferase, CK‐MB isoenzyme, CD8^+^ T‐cell count, CD4^+^/CD8^+^ ratio, or natural killer cell count (*p* > 0.05). Radiologically, the BO group showed significantly higher rates of diffuse bronchiolitis and large lobar consolidation (*p* < 0.05) (Table 2). In terms of treatment, the use of glucocorticoids, IVIG, and mechanical ventilation differed significantly between the BO and non‐BO groups (*p* < 0.05) (Table 1).

**TABLE 2:** Multivariate logistic regression analysis of risk factors for PIBO.

Variables	*β*	S. E	*Z*	*p*	OR (95% CI)
Intercept	−6.55	0.91	−7.24	< 0.001	0.00 (0.00∼0.01)
Wheezing	1.56	0.44	3.53	< 0.001	4.78 (2.00∼11.40)
Large lobar consolidation	2.14	0.44	4.92	< 0.001	8.53 (3.63∼20.02)
Mechanical ventilation	1.68	1.01	1.66	0.096	5.35 (0.74∼38.56)
Length of hospital stay	0.18	0.05	3.48	< 0.001	1.20 (1.08∼1.33)
Duration of fever	0.18	0.05	3.82	< 0.001	1.20 (1.09∼1.32)

Abbreviations: CI, confidence interval; OR, odds ratio.

### 3.2. LASSO‐Based Variable Selection and Multivariate Logistic Regression Analysis

**FIGURE 2:**
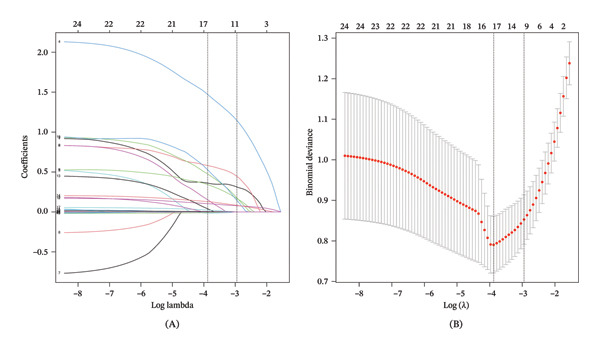
LASSO regression analysis of 25 risk factors associated with BO in pediatric patients with SAP. (A) Illustration of the coefficient profiles of the selected variables plotted against log(*λ*). (B) The relationship between the LASSO regression coefficients and the *λ* values. In each plot, the left vertical dashed line indicates the log(*λ*) value corresponding to the minimum mean squared error, while the right vertical dashed line represents the log(*λ*) value at one standard error above the minimum. The error bars denote the 95% confidence intervals.

Univariate analysis revealed 26 significant variables. To mitigate the risk of overfitting while preserving the most predictive subset, we employed LASSO regression. Notably, diffuse bronchiolitis was excluded from the analysis due to its low prevalence within the dataset. LASSO regression penalized variables, keeping only those with nonzero coefficients. It selected nine variables: PICU admission, wheezing, atopy, large lobar consolidation, respiratory failure, age, length of hospital stay, mechanical ventilation, and the duration of fever (Figure 2). These were then used as independent predictors in multivariate logistic regression analyses. These variables were subsequently used as independent predictors in multivariate logistic regression analyses.

Multivariable analysis identified the following independent risk factors for BO: wheezing (odds ratio [OR] = 4.78; 95% confidence interval [CI], (2.00∼11.40); *p* < 0.001), large lobar consolidation (OR = 8.53; 95% CI, 3.63∼20.02; *p* < 0.001), length of hospital stay(OR = 1.20; 95% CI, 1.08–1.33; *p* < 0.001), and duration of fever (OR = 1.20; 95% CI, 1.09–1.32; *p* < 0.001). Additionally, mechanical ventilation demonstrated a trend toward increased risk (OR = 5.35; 95% CI, 0.74–38.56; *p* = 0.096) and was included in the nomogram model due to its established clinical importance as a marker of severe illness and its consistent identification as a risk factor for BO in prior studies [17](d).

### 3.3. Development of the Nomogram Model

**FIGURE 3:**
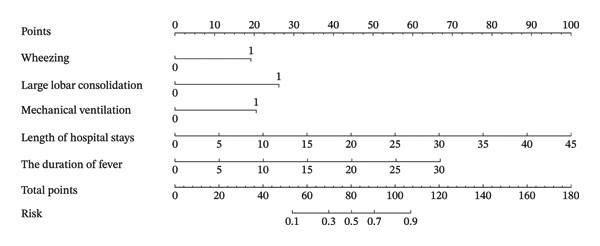
The nomogram model is designed to predict the risk of BO in pediatric patients suffering from SAP. To utilize the model, a score is determined by projecting a vertical line upward from the value of each predictor. The cumulative score is obtained by summing the individual scores of all five predictors. Subsequently, a vertical line drawn downward from the “total points” axis to the “risk of BO” axis provides the estimated probability of developing bronchiolitis obliterans. Units for each predictor are as follows: duration of fever (days), length of hospital stay (days), wheezing (yes = 1, no = 0), large lobar consolidation (yes = 1, no = 0), and mechanical ventilation (yes = 1, no = 0).

**FIGURE 4:**
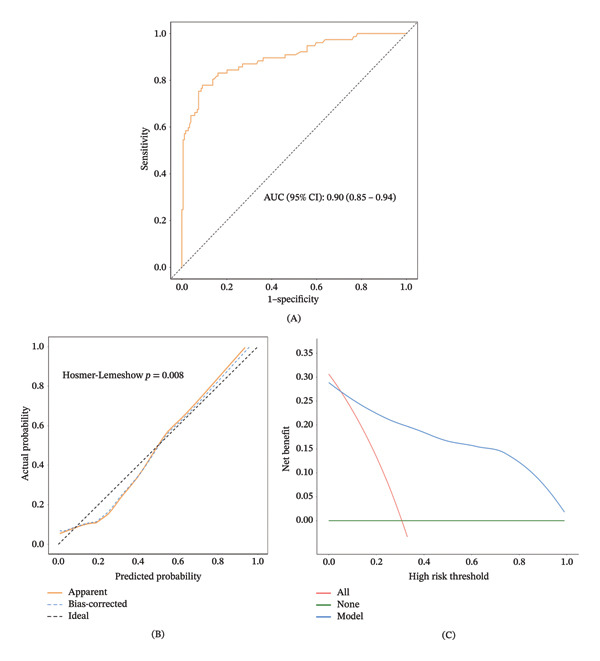
Performance evaluation of the nomogram. (A) Illustration of the area under the curve (AUC) associated with the nomogram. (B) Calibration curve of the nomogram. (C) Decision curve analysis (DCA) pertaining to the nomogram.

A predictive nomogram model for assessing the risk of BO was constructed utilizing five risk factors identified through multivariate logistic regression analyses (Figure 3). The nomogram assigns a score to each predictor; a higher total score indicates an increased risk of BO. To calculate the total score for an individual patient, points corresponding to each variable are summed after aligning their values with the point scale. The model showed a good fit as confirmed by the Hosmer–Lemeshow test (*p* = 0.019). It exhibited strong discriminative ability, with an area under the ROC curve (AUC) of 0.90 (95% CI: 0.85–0.94) (Figure 4A). The calibration curve demonstrated a close agreement between predicted and observed probabilities (Figure 4B). DCA demonstrated that, although the nomogram yielded a statistically positive net benefit compared with the “treat all” or “treat none” strategies across the threshold probability range of 0.04–1.0, the primary analysis was restricted to the clinically more actionable range of 0.10–0.70, in keeping with best practices for clinical decision analysis (Figure 4C).

## 4. Discussion

PIBO most commonly arises as a severe, long‐term pulmonary sequela of severe childhood pneumonia, with the extent of initial lung injury being the primary determinant of obliterative risk [5]. Over the past decade, the incidence of PIBO has risen worldwide, with particularly striking increases in Latin America and parts of Asia [5]. Although considerable progress has been made in understanding PIBO, the precise tools for its early detection and diagnosis are still lacking. Colom et al. [18] demonstrated that ADV is not only the primary pathogen of PIBO but also an independent risk factor for its development. Emerging evidence demonstrates that ADV‐associated PIBO accounts for 20%–69% of the total PIBO burden [19, 20]. In this study, we retrospectively enrolled 251 children with SAP and developed a concise nomogram for dynamic risk assessment during hospitalization of subsequent PIBO. Multivariate logistic regression identified wheezing, large lobar consolidation, mechanical ventilation, length of hospital stay, and duration of fever as independent risk factors associated with the development of PIBO.

The reported incidence of PIBO following SAP exhibits considerable variability, with rates ranging from 15.8% to 43.4% [10, 11, 21–23]. Our observed incidence of 30.7% is consistent with the findings of Yu et al. [23]. Such variations in reported incidence may be attributed to differences in sample size, geographic distribution, quality of medical care, and statistical methodologies employed in these studies. Compared with previous studies, we incorporated a broader set of candidate variables and employed a feature selection process involving univariate screening followed by LASSO regularization and multivariable regression. This approach efficiently selected a parsimonious set of highly predictive features, thereby optimizing model interpretability and generalizability while effectively avoiding overfitting.

Wheezing was identified as an independent risk factor for PIBO following SAP (OR = 4.78, 95% CI (2.00∼11.40) *p* < 0.001). This finding is consistent with a meta‐analysis by Liu et al., which incorporated four studies published between 1998 and 2020 comprising 104 cases and 368 controls, demonstrating a significant positive association between wheezing and PIBO, with a pooled OR of 7.73 (95% CI: 2.73–21.93) [24]. From a pathophysiological perspective, PIBO is fundamentally characterized by aberrant repair following inflammatory injury to the small airways, leading to fibrotic obliteration. This process results in narrowing, distortion, and even complete occlusion of the small airway lumen, causing fixed airway obstruction and airflow limitation. Clinically, patients present with persistent or recurrent wheezing that responds poorly to conventional bronchodilators, often accompanied by post‐exertional cough and dyspnea [25]. Unlike typical asthmatic wheezing, PIBO‐associated wheezing arises largely from irreversible fibrous narrowing of the small airways rather than reversible smooth muscle spasm, thus accounting for the poor response to bronchodilator therapy [26]. The severity and frequency of wheezing are closely correlated with the degree of obstructive ventilatory dysfunction on pulmonary function testing, primarily manifested as a reduction in forced expiratory volume in one second (FEV_1_) and a marked decrease in maximal mid‐expiratory flow (MEF_25_–_75_) [27]^.^Persistent high fever, a characteristic clinical manifestation of SAP, has been recognized by Xie et al. as a significant risk factor for SAP in children [6]. Our findings further confirm that duration of fever is an independent risk factor for PIBO following SAP, consistent with the study by Zhong et al., which demonstrated a linear association between fever duration and the risk of BO [22]. It is important to acknowledge that a definitive threshold for the duration of fever has yet to be established, and the administration of glucocorticoids may complicate the evaluation of fever duration. Our study further revealed that the length of hospital stay serves as an independent risk factor for PIBO following SAP. The length of hospital stay may be considered a proxy for the severity of pneumonia, suggesting a correlation between the severity of ADV pneumonia and the risk of developing BO. This is consistent with the findings reported by Zhang et al. [28] and Murtagh et al. [29].

In our study, the association between mechanical ventilation and BO risk demonstrated a wide CI (OR = 5.35; 95% CI: 0.74–38.56), indicating considerable uncertainty in the effect estimate. This is most likely due to the small number of patients who required mechanical ventilation and subsequently developed BO. Nevertheless, the point estimate (OR = 5.35) suggests that mechanical ventilation remains a potentially important risk factor, which is consistent with previous findings [4, 21, 29]. Mechanical ventilation often marks the extreme severity of illness and may serve as a proxy for substantial lung injury. Thus, although our study was underpowered to precisely quantify its risk due to sample size limitations, the results strongly suggest that mechanical ventilation is a key clinical warning sign for BO. Future studies with larger sample sizes are warranted to more accurately evaluate the magnitude of this risk.

Large lobar consolidation is a well‐recognized radiographic feature frequently associated with severe pneumonia. In our study, large lobar consolidation (45.42%) was the most common HRCT finding of PIBO children in the acute stage. Although it has previously been established as an independent risk factor for PIBO following MP infection [16, 30], its role in SAP‐associated PIBO had remained unclear. Our study now demonstrates that it also serves as a strong and independent predictor of PIBO following SAP. Imaging findings of diffuse bronchiolitis were associated with small‐airway pathology. Although imaging findings of diffuse bronchiolitis—which are associated with small‐airway pathology—showed statistically significant intergroup differences in the univariate analysis, this variable was excluded from the multivariate regression model due to an insufficient number of positive events. This statistical limitation precludes definitive conclusions, and future studies with larger sample sizes are warranted to validate this association. In our study, although univariate analysis revealed a significant difference in LDH levels between the BO and non‐BO groups, it was not incorporated into the nomogram model. This finding contrasts with reports by Zou et al. and Yuan et al., who consistently identified serum LDH as an independent predictor of BO following SAP [11, 31]. Regarding mixed infection, only mycoplasma co‐infection showed a statistically significant difference between the two groups but was not an independent predictor. Patients who developed BO demonstrated significantly greater use of glucocorticoids and IVIG, along with a higher burden of both pulmonary and extrapulmonary complications, indicating that more SAP may be a significant risk factor for BO.

We ultimately developed a nomogram prediction model based on wheezing, large lobar consolidation, mechanical ventilation, duration of fever, and length of hospital stay. The strengths of this model are threefold. Firstly, all predictors are bedside‐accessible routine clinical variables that do not require specialized laboratory testing, thus providing favorable applicability even in resource‐limited primary healthcare settings; secondly, it assists clinicians in the early identification of high‐risk patients, thereby facilitating timely diagnosis; thirdly, early detection and intervention guided by the nomogram may support optimized patient management and could potentially reduce the risk of PIBO in pediatric patients with SAP, thereby improving overall prognosis.

Our study has several limitations. First, its single‐center design may limit the generalizability of our findings, and validation through future multicenter studies is necessary. Second, the sample size of children with BO was relatively small, owing to the condition’s low incidence, which potentially introduces selection bias. Third, we did not perform subgroup or interaction analyses to explore variations in the risk factors’ effects among different patient groups (e.g., stratified by single versus co‐infection status). Fourth, predictors like length of hospital stay and duration of fever are not obtained at initial presentation but during hospitalization. While this limits the model’s use for early risk stratification, achieving risk stratification within weeks still significantly improves upon current clinical delays of several months. Thus, we propose this nomogram as an in‐hospital monitoring tool to facilitate earlier identification of high‐risk children.

## 5. Conclusions

Our study developed a nomogram incorporating five predictive factors—wheezing, large lobar consolidation, mechanical ventilation, duration of fever, and length of hospital stay—to estimate the risk of PIBO in children with SAP. The model demonstrated excellent predictive performance and shows potential to aid in clinical identification and treatment decision‐making for patients with PIBO following SAP.

## Author Contributions

Gang Chen: data curation, formal analysis, writing–original draft, writing–review & editing, and software. Xing Liu: data curation, formal analysis, and writing–original draft. Xiuchang Ma: formal analysis, methodology, and writing–review and editing. Jinhuan Wu: methodology and writing–review and editing. Yaowen Liang: data curation, methodology, and software. Yuchen Du: data curation. Wenxian Qian: data curation. Xiaoyu Song: data curation. Apeng Chen: writing–original draft, writing–review and editing, and supervision. Changhua Yi: data curation, formal analysis, writing–original draft, writing–review and editing, and software. Man Tian: conceptualization, project administration, supervision, and writing–review and editing.

## Funding

This work was supported by the Key Project of Medical Scientific Research Program of Jiangsu Provincial Health Commission (Grant No.: K2024022), the National Natural Science Foundation of China (Grant No.: 32273019), Internal Project of Nanjing Children’s Hospital (Grant No.: ZTG2025‐105), High‐Level Talent Research Project of the Second Hospital of Nanjing (Grant No. 0313504), and the Jiangsu Innovation and Entrepreneurship Talent Project (Grant No. JSSCRC2023569).

## Disclosure

All authors have read and approved the final manuscript.

## Consent

The authors have nothing to report.

## Conflicts of Interest

The authors declare no conflicts of interest.

## Data Availability

The datasets generated and analyzed during the current study are not publicly accessible due to the inclusion of patient data sourced from the hospital’s electronic medical record system. However, they can be obtained from the corresponding author upon reasonable request.
